# Mesenchymal Stem/Stromal Cell-Free Therapies: Challenges and Opportunities

**DOI:** 10.1007/s12033-026-01552-7

**Published:** 2026-01-25

**Authors:** Rebecca Shin-Yee Wong, Ee Wern Tan, Nancy Choon-Si Ng, Bey Hing Goh

**Affiliations:** 1 Department of Medical Education, Sir Jeffrey Cheah Sunway Medical School, Faculty of Medical and Life Sciences, Sunway University, Bandar Sunway, Selangor Malaysia; 2 Sunway Biofunctional Molecule Discovery Centre, Faculty of Medical and Life Sciences, Sunway University, Bandar Sunway, Selangor Malaysia; 3 Faculty of Health, Australian Research Centre in Complementary and Integrative Medicine, University of Technology Sydney, Ultimo, Australia; 4 Graduate Institute of Cancer Biology and Drug Discovery, College of Medical Science and Technology, Taipei Medical University, Taipei, Taiwan; 5 Faculty of Medicine, University of Cyberjaya, Cyberjaya, Malaysia

**Keywords:** Mesenchymal stem/stromal cells, Cell-free therapies, Secretome, Extracellular vesicles, Exosomes, Opportunities, Challenges

## Abstract

Mesenchymal stem/stromal stem cells (MSCs) are promising therapeutic candidates in regenerative medicine and tissue engineering. MSCs have been applied in many medical conditions and their therapeutic effects, safety, and efficacy have been well established over the past few decades. However, several challenges exist when utilizing whole cells in research and the clinical settings. As a result, researchers have turned their attention to cell-free alternatives to overcome these challenges. One promising approach that can achieve the desired therapeutic effects without the need of whole-cell transplantation is the use of MSC-conditioned medium and secretome. This article gives an overview of the advantages of using cell-free strategies over cell-based strategies and the various types of cell-free alternatives available. It also critically examines the various cell-free approaches in MSC research and therapy and provides an in-depth discussion on the opportunities and challenges of using these strategies, with an emphasis on recent advances in the field.

## Introduction

Mesenchymal stem/stromal cells (MSCs) are multipotent cells that show triploblastic differentiation potential. Under specific conditions, these cells can be differentiated into cells of endodermal-, mesodermal- and ectodermal lineages [1]. In addition to their multipotent potential, MSCs are favored therapeutic candidates for a number of advantages. For example, there are less ethical issues when using MSCs, in comparison to using embryonic stem cells, whereas MSCs are relatively easy to culture in the laboratory when compared to other stem cell types. Due to their availability in multiple locations of the body (e.g., adipose tissue, amniotic fluid, dental pulp, bone marrow, umbilical cord, etc.), harvesting of MSCs is also relatively easy. MSCs’ are cells with low immunogenic potential and are suitable to be used as autograft and allograft [2, 3].

Numerous studies have reported the safety profile and treatment efficacy of MSCs, while the number of clinical studies involving the use of MSCs is on the rise. The most common sources of MSCs used in clinical studies are bone marrow (BM)-derived MSCs, adipose tissue (AT)-derived MSCs, and umbilical cord/umbilical cord blood (UC)-derived MSCs [4]. On the other hand, MSCs have been applied in many medical conditions clinically, such as osteoarthritis [5], heart diseases [6], neurologic diseases [7], immune-related diseases [8, 9], and many others.

However, despite the well-established safety profile of MSCs and their diverse therapeutic effects, the use of cell-based therapies in research and treatment is often accompanied for several challenges. One major challenge is the heterogeneity of MSCs, which is contributed by a number of factors such as the age, gender, and health status of the donor, as well as the culture conditions of MSCs. MSCs from different sources have been shown to demonstrate differences in their phenotypical and functional properties [10]. On the other hand, the large-scale production of MSCs from primary sources can be challenging in terms of the technicality in bioprocessing and the high costs involved in MSC manufacturing [11], which needs to adhere to strict good manufacturing practice (GMP). The administration of whole cells into human subjects is subject to strict regulation and different countries have their own sets of criteria, making the approval process lengthy and challenging [11].

In view of the many challenges in cell-based therapies, researchers have explored alternatives, such as using the secretome of MSCs, which consists of soluble factors and extracellular vesicles secreted by MSCs. This review gives an overview of MSCs and their therapeutic effects, followed by a comprehensive discussion of MSC cell-free strategies. The opportunities and challenges of cell-free strategies are deliberated, with an emphasis of recent advances and consolidation of key findings in this area of research.

### Mesenchymal Stem Cells and Their Therapeutic Effects

Friedenstein and colleagues first discovered MSCs in the 1970s by observing a population of non-hematopoietic stem cells in the bone marrow with multi-lineage differentiation potential [12]. Since then, MSCs have been identified in multiple tissues and their properties have been extensively studied. The characterization of MSCs depends on at least three minimum criteria by the International Society for Cell Therapy (ISCT), namely, the ability to adhere to plastics, the presence and absence of certain surface markers and the ability to differentiate into cells of mesodermal origin (i.e., adipocytes, chondrocytes, and osteoblasts) [13]. Over the recent decades, there is an increasing number of preclinical and clinical studies on the applications of MSCs in various medical conditions owing to their diverse therapeutic benefits, making MSCs popular and promising therapeutic candidates for cell-based therapies.

Numerous studies have shown that MSCs can differentiate into cells of all three germ layers such as adipocytes, osteoblasts, chondrocytes, neurons, hepatocytes, and pancreatic beta cells when cultured under specific conditions in the laboratory [1]. Research has shown that MSCs can interact with various immune cells and exert their anti-inflammatory, immunosuppressive and immunomodulatory effects [14, 15]. Therefore, MSCs have been applied in autoimmune diseases, such as rheumatoid arthritis [16], multiple sclerosis [17], and systemic lupus erythematosus [18]. Another beneficial effect of MSCs is its ability to migrate to sites of inflammation and injury [19]. This is an important property of MSCs as it allows MSCs to move to sites where repair and regeneration are needed. Other therapeutic effects of MSCs include anti-apoptotic [20], pro-angiogenic [21], and anti-fibrotic effects [22], which have been widely studied in many medical conditions.

### Challenges of Cell-Based Therapies and Advantages of Cell-Free Alternatives

When using whole cells for clinical application, a large number of MSCs are often required. In some conditions, multiple injections may be necessary and harvesting the cells from the donor, especially in the case of an autograft, may be challenging. This is because MSCs from primary sources cannot be perpetually cultured in the laboratory. Studies have shown that senescence takes place after many passages and that the functional characteristics and differential potential of MSCs change with increasing passage number [23]. With the use of cell-free products, researchers can overcome the limited supply of MSCs and avoid repeated harvests from the donor. Research has shown that it is feasible to produce cell-free products (such as extracellular vesicles) from MSCs using a robust culture system under serum- or xeno-free conditions [24].

Another advantage of cell-free products over cell-based products is that the former bypasses some undesirable effects of the latter. Studies have shown that MSCs are capable of teratoma formation and tumorigenesis. For example, [25] demonstrated enhanced tumorigenic potential in of glioblastoma mediated by MSCs via cell–cell communications. MSCs also have been shown to aggravate tumor growth in vivo regardless of their route of administration [26]. The secretome and conditioned medium of MSCs, on the other hand, are relatively safer in this context, as they cannot differentiate into tumor cells and are less likely to support tumor growth.

Although MSCs possess homing ability to sites of injury and inflammation, studies have shown that the biodistribution of MSCs greatly depends on the route of administration. The intravenous route can result in MSC entrapment in the lungs, with most of the cells not reaching the target sites [27]. One way to minimize MSC entrapment is through injection of MSCs into selected arteries (intra-arterial route). Other routes of administration (e.g., intramuscular, subcutaneous, or tropical) are limited to the site of application. The use of conditioned medium and secretome can help to solve the problem of entrapment. It is possible to label MSC exosomes or extracellular vesicles (EV) and track their fate in vivo. Research has shown that the effects of IV injections of MSC exosomes mimic the effects of whole-cell injections. In rats with spinal cord injury, injected MSC exosomes targeted the M2-type macrophages and localized in the injured areas, instead of the non-injured areas [28].

Compared to MSCs, the cell-free derivatives have a lower immunogenicity. MSCs were traditionally considered immune-privileged cells. However, research has shown that they can be immune evasive and are no longer considered immune privileged [29]. Even though both allografts and autografts of MSCs can be used, there have been reports on adverse host immune response against transplanted MSCs. In an equine model, cytotoxic antibodies were produced after repeated intra-articular injections of allogenic synovial membrane MSCs [30]. Immune incompatibility has also been reported with the use of allograft. In a mouse model, allogenic MSCs stimulated graft rejection in the host due to major histocompatibility (MHC) mismatch [31]. The use of conditioned medium and secretome may potentially help avoid these undesirable host immune responses.

**Fig. 1:**
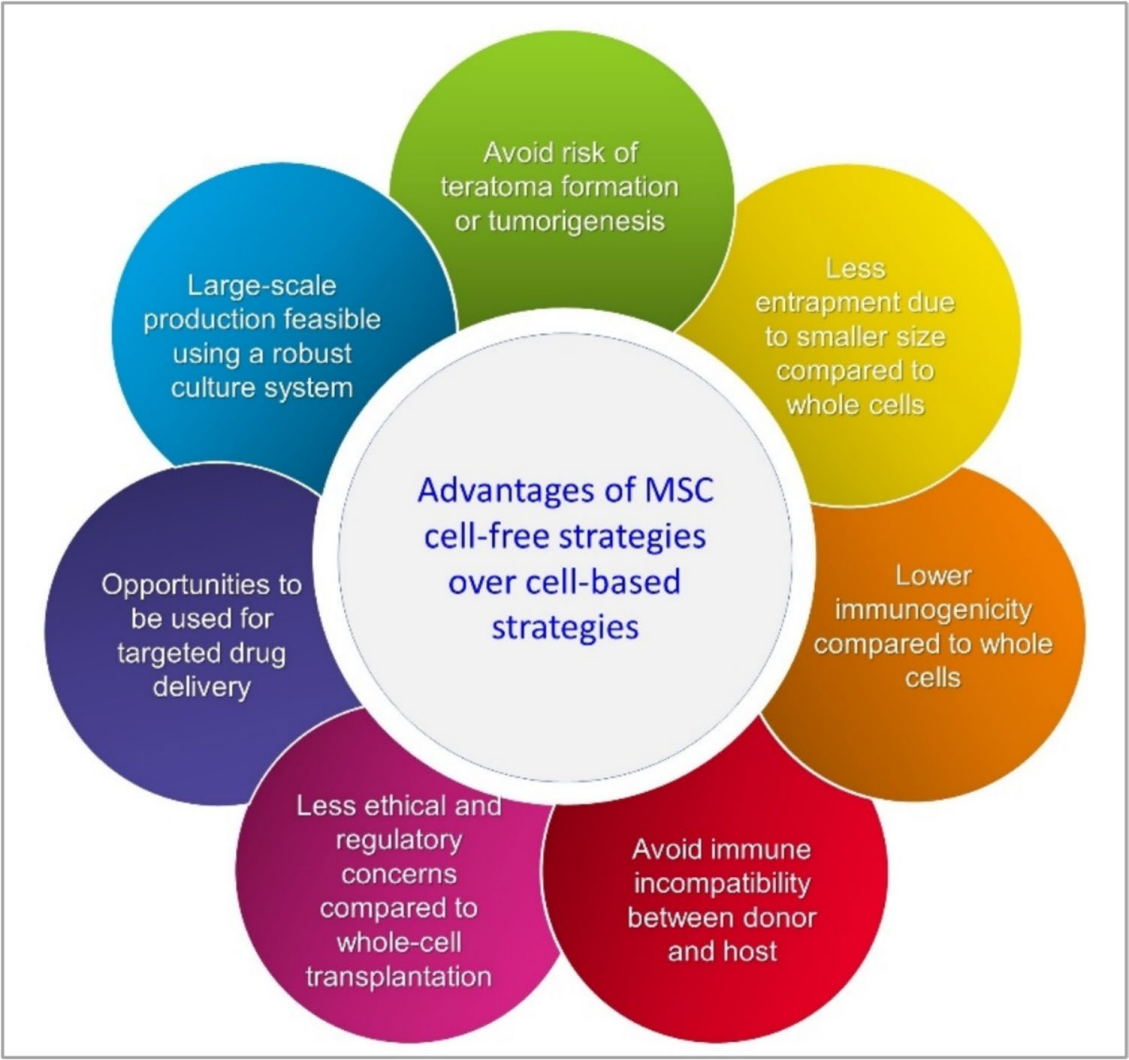
Advantages of MSC cell-free strategies over cell-based strategies

Due to the small size of exosomes, as well as their homing effect, researchers have also explored the use of MSC exosomes in drug delivery. For example, MSC exosomes loaded with various anti-cancer drugs have been used to target various cancers, such as oral squamous carcinoma [32], hepatocellular carcinoma [33], osteosarcoma [34], breast cancer [35], and pancreatic cancer [36]. Other advantages may include avoidance of ethical and regulatory issues of using whole cells in MSC-based therapy and the avoidance of transfer of infectious agents from the donor to the recipient in allogenic MSC transplantation. The advantages of cell-free strategies are summarized in Fig. 1.

### Opportunities of MSC Cell-Free Therapies

Due to the many challenges faced in MSC cell-based therapies, researchers have now turned their attention to cell-free therapies for alternative therapeutic options. This section explores the different types of cell-free alternatives and their therapeutic potential.

### Types of Cell-Free Alternatives

Recently, many studies have explored the secretome of MSC, including its composition and therapeutic potential. [37] first used the term “secretome” in their study of a eubacterium (*Bacillus subtilis*) to describe proteins secreted by bacteria and the machineries involved in these secreted proteins. The term “secretomics” is a subset of proteomics, which include information on secreted proteins and related pathways. The secretome of MSC, on the other hand, refers to the set of substances released by MSCs into their extracellular environment, which have diverse biologic functions.

**Fig. 2:**
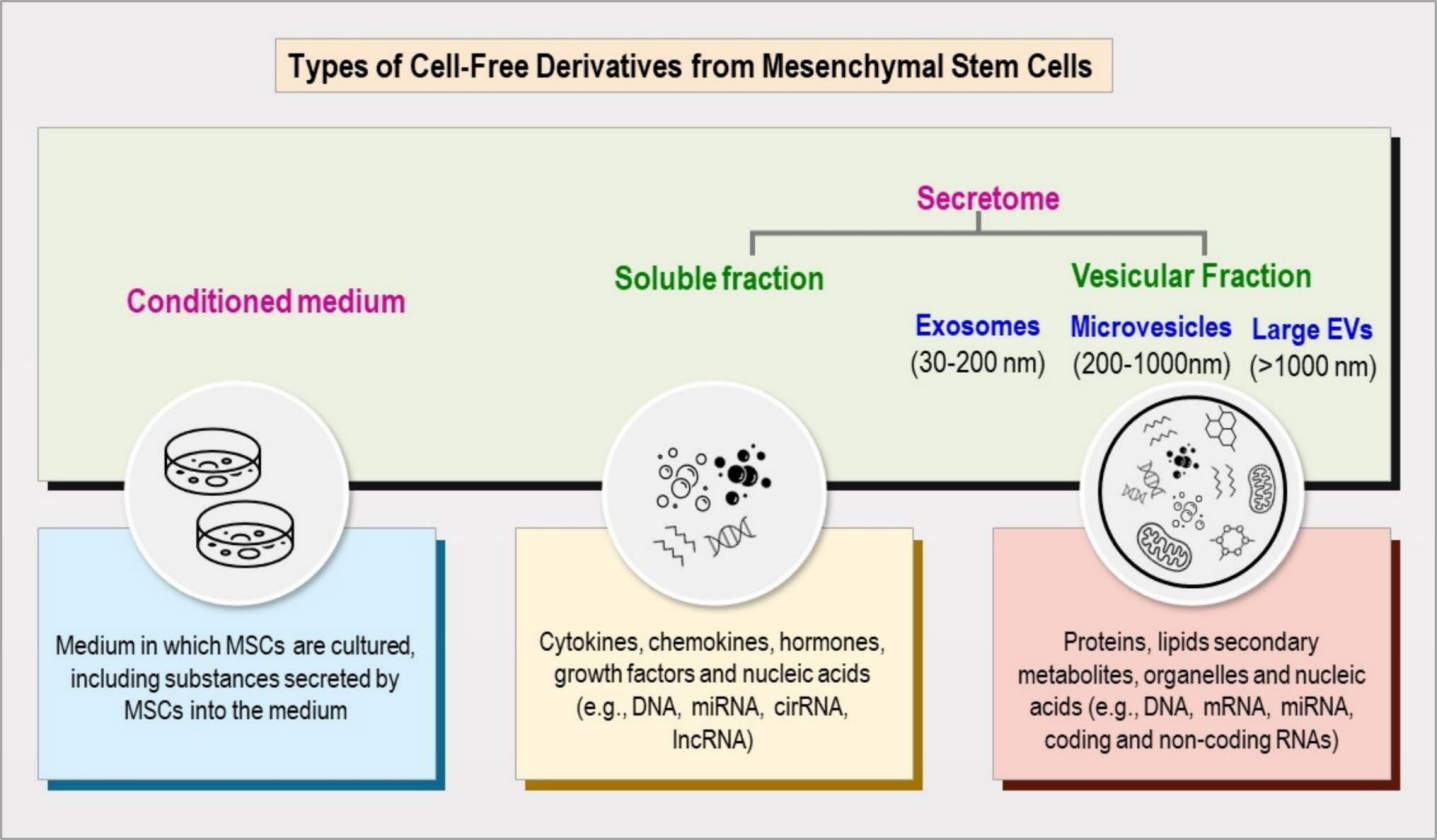
Types of cell-free derivatives from mesenchymal stem cells. *DNA *deoxyribonucleic Acid, *RNA* ribonucleic Acid, *miRNA* MicroRNA, *cirRNA* circular RNA, *lncRNA* Long non-coding RNA, *MSC*s mesenchymal stem cells

The secretome of MSC consists of two main components, namely, the soluble fraction and vesicular fraction. Cytokines, chemokines, hormones, growth factors, and nucleic acids (e.g., DNA, miRNA, cirRNA, lncRNA) are examples of MSC-derived soluble factors. The vesicular fraction consists of extracellular vesicles (EVs), which can be further divided into three categories based on size: 1) exosomes (also called nanovesicles, 30–200 nm), 2) microvesicles (also called ectosomes, 200–1000 nm) and 3) large extracellular vesicles (EVs, > 1000 nm). Apoptotic bodies and oncosomes belong to large EVs. The composition of the vesicular fraction includes proteins, lipids secondary metabolites, and nucleic acids (e.g., DNA, mRNA, miRNA, coding, and non-coding RNAs), organelles, and nuclear fractions [38–40]. Although some use the terms “secretome” and conditioned medium interchangeably, there are some differences between the two. The latter refers to the culture medium in which MSCs have been grown, which includes all the bioactive substances secreted by MSCs. The different types of cell-free derivatives are summarized in Fig. 2.

### Therapeutic Potential of MSC-Derived Soluble Factors and Conditioned Medium

In MSC-conditioned medium, the soluble fraction of the MSC secretome is made up of a vast array of soluble factors such as chemokines (e.g., eostaxin-3), cytokines [e.g., interleukin 10 (IL-10), tumor necrosis factor-*α* (TNF-*α*)], growth factors [e.g., hepatocyte growth factor (HGF), transforming growth factor-*β* (TGF-*β*)], lipids, proteases, and proteins. In addition, deoxyribonucleic acid (DNA) ribonucleic acid (RNA), circular RNA (circ-RNA) micro-RNA (miRNA) and long non-coding RNA (lncRNA) are also part of the soluble fraction [40, 41]. Studies have explored the therapeutic effects of various soluble factors. As information on MSC soluble factors in the published literature is overwhelming, only selected examples of these soluble factors and their therapeutic effects are summarized in Table 1.

**Table 1:** Summary of therapeutic effects of MSC-conditioned medium and soluble factors

Therapeutic effect	Soluble factors	Remarks	References
Anti-apoptotic effects	HGF	Reduction in apoptotic rate with application of BM-MSCs overexpressing HGF in mouse model with radiation-induced lung injury	[42]
	HGF	BM-MSCs exerted anti-apoptotic effects in a mouse model of elastase-induced emphysema via HGF	[43]
	VEGF	In vitro studies showed inhibition of apoptosis in cardiomyocytes mediated via VEGF from BM-MSCs in a rat model of myocardial infarction	[44]
	FGF	Inhibition of apoptosis and stimulation of proliferation in AD-MSCs from type 2 diabetes patients	[45]
Anti-fibrotic and anti-inflammatory effects	IL1RA	MSCs exerted anti-inflammatory and anti-fibrotic effects mediated by IL1RA, in bleomycin-induced lung injury in vitro and in vivo	[46]
Anti-fibrotic effects	Decorin	Conditioned medium of decorin-expressing AD-MSCs exhibited inhibitory effects on muscle fibrosis using C2C12 myoblast cells	[47]
Antimicrobial effects	AMPs	In vitro studies showed MSCs produced AMPs such as cathelicidin LL-37, hBD2, hepcidin, and LcN with antimicrobial activities against *Staphylococcus aureus* Activated MSCs administered in mice reduced number of bacteria at wound site and promoted wound healing	[48]
	hCAP-18/LL-37	Conditioned medium and MSCs showed inhibition of bacterial growth in vitro and in vivo, mediated by hCAP-18/LL-37 derived from BM-MSCs	[49]
Antioxidant effects	HO-1	Transduction of HO-1 into BM-MSCs improved inflammation and oxidative damage in PEVCs in vitro and in acute lung injury animal models	[50]
Immunomodulatory effects on macrophages	IL1RA	IL1RA secreted by BM-MSCs promoted macrophage polarization toward immunosuppressive M2 phenotype in vitro Inhibition of B cell differentiation in vivo	[51]
Immunomodulatory effects on monocytes	IL-6, HGF	UC-MSCs secreted IL-6 and HGF exerted immunosuppressive effects on monocyte differentiation to DCs Monocytes differentiated into cell types other than DCs	[52]
Immunomodulatory effects on B cells	IDO	INF-γ-primed AT-MSCs showed increased expression of IDO and suppressed proliferation of B cells and their IgG production in vitro	[53]
Immunomodulatory effects on T cells	IL-25; PD-L1	Human MSCs derived IL-25 upregulated expression of PD-L1 and exert immunosuppression on Th17 cells in vitro and in vivo via the IL-25/STAT3/PD-L1 pathway and	[54]
Immunomodulatory effects NK cells	IDO, PGE2	IDO and PGE2 released by MSCs inhibited NK cell proliferation, cytotoxicity and cytokine production, and downregulated surface expression of activating receptors on NK cells in vitro	[55]
Proangiogenic effects	VEGF	Conditioned medium of equine PB-MSCs promoted angiogenesis in vitro, which was mediated by paracrine effect of VEGF	[56]
	FGF-2	Conditioned medium of hGMSCs overexpressing FGF-2 were shown to enhance angiogenesis in vitro using HUVECs FGF-2 stimulated increased expression of growth factors (e.g., PLGF, SCF, VEGFR2) related to angiogenesis in HUVEs	[57]

*AMPs* antimicrobial peptides and proteins, *AT-MSCs/AD-MSCs* adipose tissue-derived MSCs, *BM-MSCs* bone marrow-derived MSCs, *FGF* fibroblast growth factor, *hBD2* beta-defensin, *hCAP-18/LL-37* human cathelicidin antimicrobial peptide, *HGF* hepatocyte growth factor, *hGMSCs* human Gingival MSCs, *HO-1* heme oxygenase 1, *HUVECs* human umbilical vein endothelial cells, *IL1RA* interleukin 1 receptor Antagonist, *IL* Interleukin; *INF* interferon, *LcN* Lipocalin, *MSC* mesenchymal stem Cell, *NK* natural killer, *PB-MSCs* peripheral blood-derived MSCs, *PD-L1* programmed death ligand 1, *PEVCs* pulmonary microvascular endothelial cells, *PGE2* prostaglandin E2, *PLGF* placenta growth factor, *SCF* stem cell factor, *UC-MSCs* umbilical cord-derived MSCs, *TGF-β* transforming growth factor-beta, *VEGF* Vascular endothelial growth factor, *VEGFER2* vascular endothelial growth factor receptor 2

### Therapeutic Potential of MSC Extracellular Vesicles

The EVs include exosomes, microvesicles, and large EVs. Selected preclinical and clinical studies in the past ten years showing the wide range of therapeutic effects of EVs and exosomes in various diseases are summarized in Table 2.

**Table 2:** Application of MSC extracellular vesicles/ exosomes in preclinical and clinical studies

Disease category	Disease	MSC type	Secretome components	Therapeutic effect	Type of study	References	
Preclinical studies							
Cancer	Breast cancer	BM-MSCs	Exosomes	Cytotoxic and anti-tumor effects via drug delivery	In vitro and mouse model	[35]	
	Osteosarcoma	BM-MSCs	Exosomes	Enhanced cellular uptake of drugs and anti-tumor effects via drug delivery	In vitro	[34]	
	Drug-resistant oral squamous cell carcinoma	MSCs	Exosomes	Anti-apoptotic effects and tumor suppression via drug delivery	In vitro and mouse model	[32]	
	Pancreatic cancer	BM-MSCs	Exosomes	Anti-tumor effects via drug delivery	In vitro and mouse model	[36]	
	Hepatocellular carcinoma	BM- MSCs	Exosomes	Anti-tumor effects, enhancement of repair of damaged liver cells and suppression of liver cell oxidation via drug delivery	In vitro and mouse model	[33]	
	Bladder and prostate cancers (cell lines)	AT-MSCs	Exosomes	Apoptotic, anti-proliferative and anti-angiogenic effects	In vitro	[58]	
	Renal cancer (cell line)			No significant effects			
Cardiovascular/ pulmonary conditions	Myocardial infarction	BM-MSCs	Exosomes	Enhancement of myocardial repair and cardiac function	Rat model	[59]	
	Myocardial infarction	rBM-MSCs	Exosomes	Proangiogenic and cardioprotective effects	Rat model	[60]	
	Pulmonary hypertension	hUC-MSCs	Exosomes	Attenuation of pulmonary vascular remodeling	Rat model	[61]	
	Retinal ischemia–reperfusion injury	GMSCs	Exosomes	Anti-inflammatory and neuroprotective effects	In vitro and mouse model	[62]	
Fibrosis and wounds	Diabetic nephropathy	BM-MSCs	Exosomes	Anti-fibrotic effects and improvement in renal function	Rat model	[63]	
	Chronic diabetic wound	hUC-MSCs	Exosomes	Wound healing and skin regeneration	Rat model	[64]	
	Liver cirrhosis	T-MSCs	Extracellular vesicles	Anti-fibrotic effect and inhibition of hepatic stellate cells	Invitro and mouse model	[65]	
	Skin hypertrophic scar fibrosis	AD-MSCs	Exosomes	Anti-fibrotic effects	In vitro and mouse model	[66]	
	Diabetic ulcers	AD-MSCs BM-MSCs	Extracellular vesicles	Proangiogenic effect BM-MSCs Enhanced proliferation of cells involved in wound healing	In vitro and mouse model	[67]	
	Epidermal wound	AD-MSCs	Extracellular vesicles	Enhanced epidermal regeneration via secretome effects on keratinocyte functions	Tissue-mimetic 3D hydrogel system	[68]	
Immune-related conditions	Acute graft-versus-host disease	BM-MSCs	Exosomes	Anti-inflammatory effects and prolonged survival	Mouse model	[69]	
	Rheumatoid arthritis	BM-MSCs	Extracellular vesicles	Anti-inflammatory effects	In vitro	[70]	
	Inflammatory bowel disease		Exosomes	Restoration of mucosal barrier repair and intestinal immune regulation	Mouse model	[71]	
	Osteoarthritis	hUC-MSCs	Extracellular vesicles	Anti-inflammatory and immunomodulatory effects	In vitro and rat model	[72]	
	Systemic lupus erythematosus	hUC-MSCs	Extracellular vesicles	Immunoregulatory effects	In vitro	[73]	
	Acute steroid-refractory graft-versus-host disease	BM-MSCs	Extracellular vesicles	Suppression of GVHD systems via immunomodulatory effects in vivo	GVHD mouse model	[74]	
Neurologic conditions	Perinatal brain injury	hWJ-MSCs	Exosomes	Reduction of neuroinflammation mediated by microglia	In vitro and rat model	[75]	
	Alzheimer’s disease	hBM-MSCs	Exosomes & microvesicles	Immunomodulatory and neuroprotective effects	Mouse model	[76]	
	Ischemic stroke	AD-MSCs	Extracellular vesicles	Neuroprotective effects	Mouse model	[77]	
	Spinal cord injury	BM-MSCs	Extracellular vesicles	Promotion of neurogenesis	Rat model	[78]	
	Multiple sclerosis	Rhesus monkey MSCs	Exosomes	Promotion of remyelination and reduction of neuroinflammation	Mouse model	[79]	
Disease category	Diseases	MSC type	Component of secretome	Therapeutic effect	Type of study	Reference	
Clinical studies							
Renal conditions	Stage III and IV chronic kidney disease	UC-MSCs	Extracellular vesicles	Anti-inflammatory effects and improvement of renal functions	Phase II/III clinical pilot study (*n* = 40)	[80]	
Pulmonary conditions	Severe COVID-19	Allogenic BM-MSCs	Exosomes	Oxygenation restoration, cytokine storm downregulation, and immunity reconstitution	Prospective non-randomized open-label cohort study (*n* = 24)	[81]	
	Chronic obstructive pulmonary disease	PL-MSCs	Exo-d-MAPPS	Anti-inflammatory effects and improvement of pulmonary functions	COPD mouse model and clinical study (*n* = 30)	[82]	
	Severe COVID-19	AD-MSCs	Exosomes	Resolution of pulmonary lesions on CT imaging	Phase 2a single-arm, open-labeled, interventional trial (*n* = 7)	[83]	
	Mild COVID-19	UM-MSCs	Exosomes	Resolution of pulmonary lesions on CT imaging and reduction of hospitalization duration	Pilot clinical trial (*n* = 7)	[84]	
Gastro-intestinal condition	Complex perianal fistula	P-MSCs	Exosomes	Resolution of abnormal tracts and reduced discharge via immunomodulatory effects	Phase I clinical trial (*n* = 11)	[85]	

*AD-MSCs/AT-MSCs* adipose-derived/adipose tissue-derived MSCs, *BM-MSCs* bone marrow-derived MSCs, *COVID-19* coronavirus disease 2019, *CT* computed tomography, *Exo-d-MAPPS* exosome-derived multiple allogeneic protein paracrine signaling, *GMSCs* Gingiva-derived MSCs, *GVHD* graft-versus-host disease, *P****/****PL-MSCs* placenta/placental tissue-derived MSCs, *T-MSCs* tonsil-derived MSCs, *UC-MSCs* umbilical cord-derived MSCs, *WJ-MSCs* Wharton’s Jelly-derived MSCs

Early clinical experience supports biologic activity and feasibility of MSC cell-free therapies in humans. In CKD, UC-MSC-EVs were associated with improved renal function and inflammatory profiles in a pilot phase II/III cohort [80]. In respiratory disease, exosome products have been explored via nebulization/inhalation and systemic delivery, with reports of improved oxygenation, radiologic resolution, and symptom duration in COVID-19 cohorts [81, 83, 84], and exploratory functional gains in COPD linked to anti-inflammatory effects [82]. In refractory perianal fistula, local injection of placenta-MSC-derived exosomes achieved tract resolution and reduced discharge in a phase I setting [85]. Although these studies are small and often uncontrolled, they demonstrate route flexibility, favorable tolerability, and disease-appropriate endpoints that can anchor upcoming randomized trials.

### Mechanisms Underpinning MSC Cell-Free Therapeutic Effects

MSC-derived conditioned medium and extracellular vesicles (EVs) exert multi-layered effects that are primarily paracrine and immunomodulatory rather than replacement-based. First, immune re-programming: MSC secretome polarizes macrophages from pro-inflammatory M1 to reparative M2 phenotypes (e.g., IL-1RA, PGE2, IDO), suppresses Th17 responses via IL-25/STAT3-mediated PD-L1 upregulation, and inhibits dendritic-cell maturation through IL-6/HGF [51–55]. Second, anti-inflammatory/anti-fibrotic signaling: soluble IL-1RA and EV miRNAs attenuate NF-κB activity and TGF-β/Smad profibrotic cascades, reducing fibroblast activation and ECM deposition [46, 66]. Third, pro-angiogenic cues: EV cargo (VEGF, FGF-2; pro-angiogenic miRNAs) activates VEGFR2/ERK and PI3K/Akt pathways in endothelial cells, enhancing sprouting and perfusion [56, 57]. Fourth, cytoprotection and apoptosis resistance: factors such as HGF, VEGF and EV-borne miRNAs modulate PI3K/Akt and Bcl-2 family signaling to limit caspase activation in stressed tissues [42–45]. Fifth, oxidative-stress buffering: HO-1 augmentation and downstream Nrf2-responsive programs mitigate ROS-driven injury [50]. Sixth, barrier and tissue repair effects: EVs transfer regulatory RNAs and proteins that preserve epithelial/endothelial junctions and promote keratinocyte and neural precursor migration and maturation [68, 75–79]. Finally, antimicrobial actions: constitutive or induced secretion of LL-37 and other AMPs curtails bacterial growth and modulates innate immune effector cells [48, 49]. Collectively, these mechanisms align with disease-specific phenotypes as summarized in Tables 1 and 2 and explain why cell-free products can phenocopy many benefits of whole-cell MSC therapy.

### Clinical Trial Landscape for MSC Cell-Free Products

To contextualize translation timelines, we summarized registered and published trials of MSC-derived EVs/exosomes and MSC-conditioned medium (CM) across ClinicalTrials.gov, EU-CTR, ChiCTR, and related registries. The most active indications cluster in respiratory disease (e.g., COVID-19/COPD), renal disease (CKD), fistulizing disorders, and dermatologic/wound repair, consistent with signals seen in Table 2 [80–85]. Trials span early feasibility (pilot, phase I) to small phase II designs, with routes including intravenous, nebulized/inhaled, intralesional, and topical delivery. Common primary endpoints are safety (AEs/SAEs, coagulation parameters), feasibility, and exploratory efficacy (imaging, organ function indices, inflammatory biomarkers). While safety profiles are encouraging to date, heterogeneity in product characterization (EV dose, particle definition, potency assays) and small sample sizes limit meta-analytic inference. Harmonized manufacturing and release criteria [86] remain critical gating factors for progression to pivotal trials Table 3

**Table 3:** Ongoing and completed clinical trials of MSC-derived exosomes or conditioned medium

Registry ID	Indication	Product (EVs/CM; source)	Route/dose	Phase	Sample size	Primary endpoint	Status	Key notes	References
–	Chronic kidney disease (Stage III–IV)	EVs; UC-MSC	IV (wk 1) + intra-arterial (wk 2); ~ 100 µg/kg per dose	Pilot Phase II/III	40	eGFR, creatinine, UACR, inflammatory markers	Completed/Published	Significant renal function improvement over 12 months; cytokine profile shift	[87]
–	Severe COVID-19 with ARDS	Exosomes; BM-MSC (“ExoFlo”)	IV; single course (safety/feasibility study)	Cohort (non-randomized)	24	Safety, oxygenation, and clinical recovery indices	Completed/Published	Open-label cohort; signals for improved oxygenation and cytokine modulation	[81]
–	COPD	Exo-d-MAPPS (exosome-derived paracrine product); placental MSC	NR (parenteral; see study)	Small clinical cohort	30	Safety, pulmonary function, & inflammation	Completed/Published	Combined preclinical + clinical program reporting anti-inflammatory effects	[82]
–	Severe COVID-19	Exosomes; AD-MSC	Nebulized inhalation	Phase 2a (single-arm)	7	Safety, CT lesion resolution	Completed/Published	Pilot showing radiologic improvement with inhaled EVs	[83]
–	Mild COVID-19	Exosomes; UC-MSC	Nebulized inhalation	Pilot	7	CT lesion change; hospital stay	Completed/Published	Feasibility and symptom/radiology signals	[84]
–	Complex perianal fistula (non-Crohn’s)	Exosomes; placenta-MSC	Local intralesional injection	Phase I	11	Safety; fistula tract closure	Completed/Published	Early safety and closure outcomes in non-Crohn’s cases	[85]
NCT04602104	ARDS	Exosomes; allogeneic MSC-Exos	Aerosol inhalation; Particles: 2.0 × 10^8 / 8.0 × 10^8 / 1.6 × 10^9 daily × 7 (dose escalation then randomized)	Phase 1/2	169	Safety, MTD; clinical efficacy vs. saline	Ongoing (registered)	Detailed dose schema reported in peer-reviewed summary of the registry record	[87]
NCT04356300	MODS after acute Type-A aortic dissection repair	Exosomes; MSC-Exos	IV; 150 mg daily × 14 days (prevention and treatment arms)	Randomized (two-part)	~ 60	Safety; SOFA score and complications	Ongoing (registered)	Two-part design: prophylaxis and treatment cohorts vs. SOC	[87]
NCT03437759	Large/refractory macular hole	Exosomes; MSC-Exos	Local ocular injection; 20 µg or 50 µg	Interventional	44	Visual acuity (BCVA), OCT metrics	Ongoing (registered)	Dosing and assessments specified in review’s table from registry	[87]

### Challenges of MSC Cell-Free Therapies

Although there are many advantages for using MSC-EVs and exosomes, researchers face several challenges when using these cell-free alternatives. These challenges exist at multiple levels such as (1) in vitro culture conditions, (2) methods of isolation and purification, (3) storage and maintenance, (4) safety, and (5) administration and biodistribution [88, 89].

### In Vitro Culture Conditions

One of the keys to the large-scale production of EVs is large-scale production of MSCs. This, in turn, requires long-term maintenance of MSCs to produce sustainable amounts of EVs. However, studies have shown that MSC phenotypical and functional characteristics change with increasing passage number. Senescent is observed after multiple passages and this can lead to altered therapeutic effects of the EVs produced by senescent MSCs [23]. Besides, large-scale production of MSCs is often labor intensive, expensive, and requires a large number of primary MSCs from the donor. This is especially true when multiple doses of EVs are needed for certain clinical conditions. The use of traditional culture flasks in mass production of MSCs and MSC-derived EVs is not feasible, while the use of three-dimensional culture bioreactors can greatly increase the surface area [90].

In the large-scale production of EVs, factors such as cellular confluence, passage number of MSCs, composition and serum content of culture medium, as well as the oxygen concentration, can affect the quality and quantity of EVs [91]. For the clinical application of EVs, xeno-free and serum-free medium is preferred. However, the use of xeno-free and serum-free medium may alter the properties of the MSCs and their EVs. [92] reported a xeno- and serum-free method using pooled human platelet lysate (pHPL) instead. The study reported that BM-MSCs cultured in 10% pHPL-based EV-depleted medium were able to retain their morphology, viability, surface markers, and differentiation potential and their EVs showed well-defined patterns of protein characteristics. However, a change in the medium’s pHPL content resulted in altered MSC properties and RNA profile of their EVs. The findings of this study, therefore, implicate that standardization in MSC culture is important in the large-scale production of EVs to minimize batch-to-batch variability.

### Isolation and Purification Methods

Filtration, ultracentrifugation, and affinity separation are common EV isolation methods. Usually, a combination of isolation and purification methods is necessary to obtain high-quality clinical-grade EVs. Each method of isolation and purification has its pros and cons in terms of time, purity, cost, and the degree of EV integrity. Some methods such as sequential ultracentrifugation and ultrafiltration are relatively less expensive. However, these methods are time consuming and may cause damage to exosomal integrity. Other methods such as size-exclusion chromatography is relatively fast and gives high purity but requires the use of expensive equipment and involves complex multi-step procedures. On the other hand, polymer precipitation is suitable for small- and large-sized samples but can be time consuming and has the disadvantages of protein aggregation and polymer contamination [89].

Another challenge in the isolation and purification of EVs is the presence of contaminants such as lipids, peptides, proteins, and cell debris [93]. [94] compared the quality and efficiency of various methods of EV isolation methods using nana-flow cytometry and reported that the degree of purity and amounts of contaminants present in the EVs depend on the isolation methods. In addition, there is no standardized protocol in EV isolation and purification. This results in heterogeneity in the isolated EVs. There is also a lack of scalable techniques in EV isolation. Research has shown that the use of ultracentrifugation followed by size-exclusion chromatography enhanced the yield of EVs while preserving the functional and biophysical properties of EVs [95]. However, more studies are needed to explore techniques that allow large-scale isolation and purification of EVs without damaging their integrity.

### Storage and Maintenance

The storage of EVs and exosomes is a critical step in their clinical application. The number of freeze–thaw cycles should be minimized, as repeated freezing and thawing of EVs can lead to altered integrity and functionality of EVs and exosomes. EVs and exosomes should be stored at optimal conditions to avoid deterioration of their quality and therapeutic potential, as they are sensitive to factors such as temperature, pH, and light. Generally, EVs and exosomes should be stored at − 80 C in a dark place. A change in the storage temperature can lead to variations in EV size and composition, and greater change is observed when they are stored at higher temperatures as compared to a storage temperature of − 80 C [96]. However, storage at low temperatures and repeated freeze–thaw cycles may lead to cryodamage, exosomal swelling, and exosomal aggregation. In addition, the uptake of exosomes is better at storage pH values of 4 and 10, rather than pH 7 [97]. Studies have shown that the addition of cryoprotectants (e.g., trehalose) can protect exosomes from cryodamage and aggregation [98].

### Safety

Since exosomes and viruses are similar in size, it is possible that the exosomal fraction contains contaminants like viral products and virions, as well as toxins and bacterial vesicles. However, the larger-sized microbes and fungi are less likely to be present in the exosomal fraction. Retroviral infections (e.g., HIV and HTLV-1) can alter the biogenesis of exosomes. Exosomes from infected cells have been shown to play a role in promoting infection and inflammatory responses, as the exosomes serve as natural biocarriers for the virus to spread inside the host’s body. Exosomes from the infected cells may contain nucleic acid and proteins from the virus, which may alter recipient cell functions [99]. If exosomes are extracted from an infected person and used in allogeneic transplantation, the recipient may be adversely affected by administration of exosomes produced by the infected cells.

MSCs and their EVs have been shown to possess procoagulant activities. Therefore, thrombosis is one of the safety concerns when EVs are administered in patients. Research has found that the procoagulant effects of MSCs and their EVs are related to the presence of tissue factor and phosphatidylserine, whereas a higher thrombotic risk is associated with the larger EVs [100]. In addition, it is believed that the risk of thrombosis correlates with the concentration of EVs used. Another safety concern is adverse immune reactions against the administered EVs. Although EVs and exosomes have low immunogenicity in general, [101] reported that exosomes were taken up by antigen presenting cells (APC) and elicited T-cell responses in vitro, whereas mice exposed to allogeneic exosomes were sensitized to alloantigens.

### Administration and Biodistribution

One of the challenges in the administration of EVs in human subjects is the quality control and standardization of products. The quality of EVs can be affected by many factors during bioprocessing. The consistency and reproducibility of EVs and their therapeutic effects are important factors to be considered before they are injected in human subjects. The development of reference materials and validated assays is essential to ensure the reliability of preclinical and clinical studies. Technically, it is challenging to obtain EV fractions that are completely free from the non-vesicular components. As such, the International Society for Extracellular Vesicles (ISEV) has provided a minimal set of criteria with regards to the biophysical, biochemical and functional standards for the use of EVs [86]. Categorization of EV-based therapeutics plays a crucial role in fulfilling regulatory requirements, as the categorization determines the subsequent manufacturing requirements for the translation of EVs in clinal therapies.

**Fig. 3:**
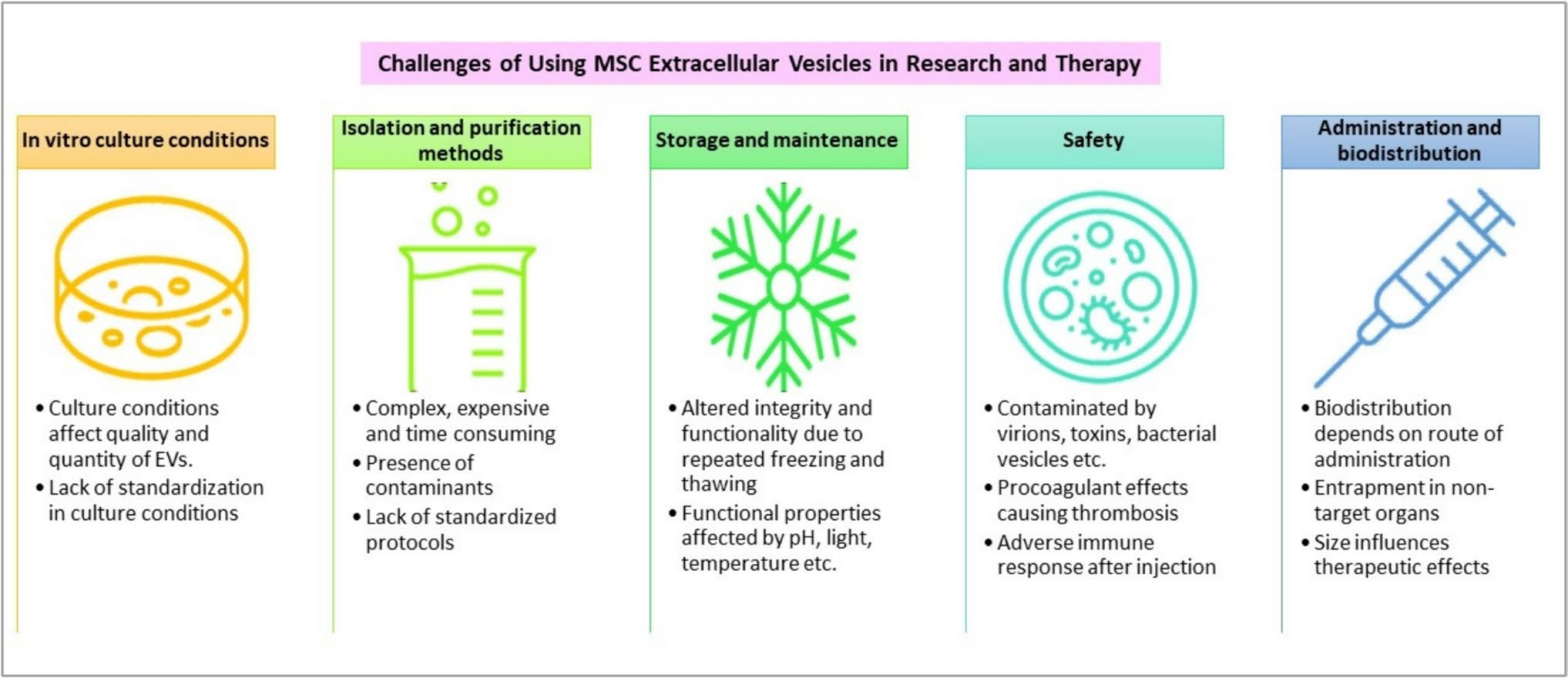
Challenges of using MSC extracellular vesicles in research and therapy. *EV* extracellular vesicles

The biodistribution of EVs is influenced by the dose, route of administration, and cell source [102]. Different routes of administration of EVs such as intravenous (IV), intraperitoneal (IP), oral, and intranasal (IN) have been reported, depending on the intended target tissues and desired therapeutic effects. In systemic injections, EVs can be entrapped in the lungs, gastrointestinal tract, liver, and spleen [102]. The size of MSC-EVs can also influence their therapeutic effects. For example, [103] demonstrated the larger MSC-EVs had better regenerative potential than smaller EVs in acute kidney injury. The difference in regenerative potential was mainly due to differences in molecular composition (e.g., mRNAs and proteins) of the EVs. The challenges of using cell-free alternatives such as MSC extracellular vesicles in research and therapy are summarized in Fig. 3.

## Conclusion

For many decades, MSCs have been regarded as promising therapeutic candidates owing to their numerous therapeutic effects. Recent studies have shown that many therapeutic effects of MSCs can be attributed to their secretome. The future of MSC research and therapy is likely to be dominated by MSC cell-free strategies, given the many advantages of MSC cell-free alternatives over cell-based therapeutic options. However, the use of cell-free alternatives is not without challenges. The fate of MSC-EVs largely depends on the scalable production of EVs that are safe, consistent in their functional properties and free from infectious contaminants. Currently, several hurdles exist when using MSC-EVs and other cell-free derivatives. The heterogeneity of MSC-EVs is multifactorial, which can be due to various factors along the manufacturing process. For example, there are no standardized protocols for MSC culture and expansion, as well as EV isolation, storage, and maintenance. Different cell source, dosing, and administration routes can also lead to variations in the therapeutic potential of MSC-EVs.

Future directions of MSC cell-free therapies should include ways to standardize the bioprocessing of MSC-EVs and other cell-free derivates, including the development of standardized protocols for processes from bench to bedside. Where EVs are applied in the clinical settings, implementation of strict quality control measures and compliance of good manufacturing practice (GMP) standards are necessary. Improvements in the scalable production of clinical-grade cell-free derivatives are also needed for the efficient use of MSC-EVs. Currently, there is a scarcity of published clinical trials on the use of EVs and the number of patients involved in the few published trials was small. Hence, more large-scale clinical trials are required to establish the safety and efficacy of MSC-EVs and cell-free derivates in human subjects. Other areas of research which may be of interest to researchers include further exploration of targeted delivery of MSC-EVs and exosomes, use of MSC-EVs for drug delivery and use of MSC-EVs in combination therapy such as combining MSC-EVs with other therapeutic agents.

## Data Availability

Data are available upon request by contacting the corresponding author.
